# An Unusual Cause of Dysphagia in a 16-Year-Old Girl

**DOI:** 10.34172/mejdd.2025.445

**Published:** 2025-10-31

**Authors:** Samira Saeian, Saeedeh Esmaeili, Mohammad Hossein Anbardar, Alireza Dehghan, Kamran B Lankarani

**Affiliations:** ^1^Gastroenterology and Hepatology Research Center, Shiraz University of Medical Sciences, Shiraz, Iran; ^2^Department of Pathology, Shiraz University of Medical Sciences, Shiraz, Iran; ^3^Medical Imaging Research Center, Shiraz University of Medical Sciences, Shiraz, Iran; ^4^Health Policy Research Center, Shiraz University of Medical Sciences, Shiraz, Iran

**Keywords:** Dysphagia, Pseudoachalasia, Leiomyoma

## Abstract

This report presents the case of a 16-year-old girl with progressive dysphagia and weight loss over 6 months. Initial examinations, laboratory findings, and upper endoscopy were unremarkable. High-resolution esophageal manometry was suspicious of achalasia, but subsequent imaging revealed the presence of a non-homogeneous mass in the esophageal wall, identified as esophageal leiomyoma through histopathological and immunohistochemical analyses. Despite the rarity of this tumor in adolescents, our findings emphasize the importance of considering esophageal leiomyoma in the differential diagnoses of intramural esophageal lesions. The patient was advised of surgical treatment, which she and her family refused. This case underscores the need for accurate tissue diagnosis via endoscopic ultrasonography (EUS)-guided biopsies to guide treatment decisions, especially in young patients presenting with significant esophageal symptoms.

## Introduction

 Esophageal leiomyoma is a rare benign tumor originating from mesenchymal cells, accounting for the majority of non-malignant esophageal neoplasms.^1,2^ Although typically diagnosed in middle-aged adults, its occurrence in adolescents is exceedingly uncommon, often leading to diagnostic challenges. Most patients remain asymptomatic, with tumors discovered incidentally during imaging or endoscopic procedures. However, when symptoms do arise, they commonly include dysphagia, retrosternal pain, and weight loss—manifestations that may mimic other esophageal disorders such as achalasia or malignancy.^1,2,3^

 Accurate diagnosis is crucial, particularly in younger patients, where invasive procedures carry greater implications. Endoscopic ultrasonography (EUS) combined with fine needle biopsy (FNB) has emerged as a minimally invasive and highly effective method for tissue characterization, enabling differentiation from other intramural lesions.^2^ This case report highlights a rare presentation of esophageal leiomyoma in a 16-year-old girl, emphasizing the importance of considering this entity in the differential diagnosis of pseudoachalasia and intramural esophageal masses even in adolescents.

## Case Report

**Figure 1:**
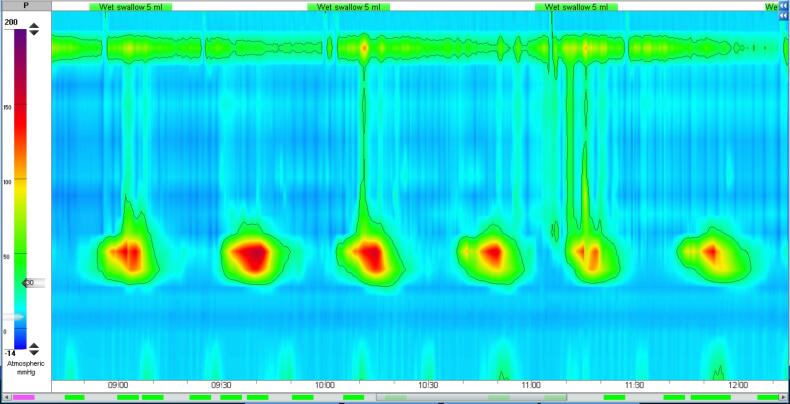
High-resolution esophageal manometry detected the absence of peristalsis with panesophageal pressurization, but LES had repeatedly very high pressure with sudden drops

**Figure 2:**
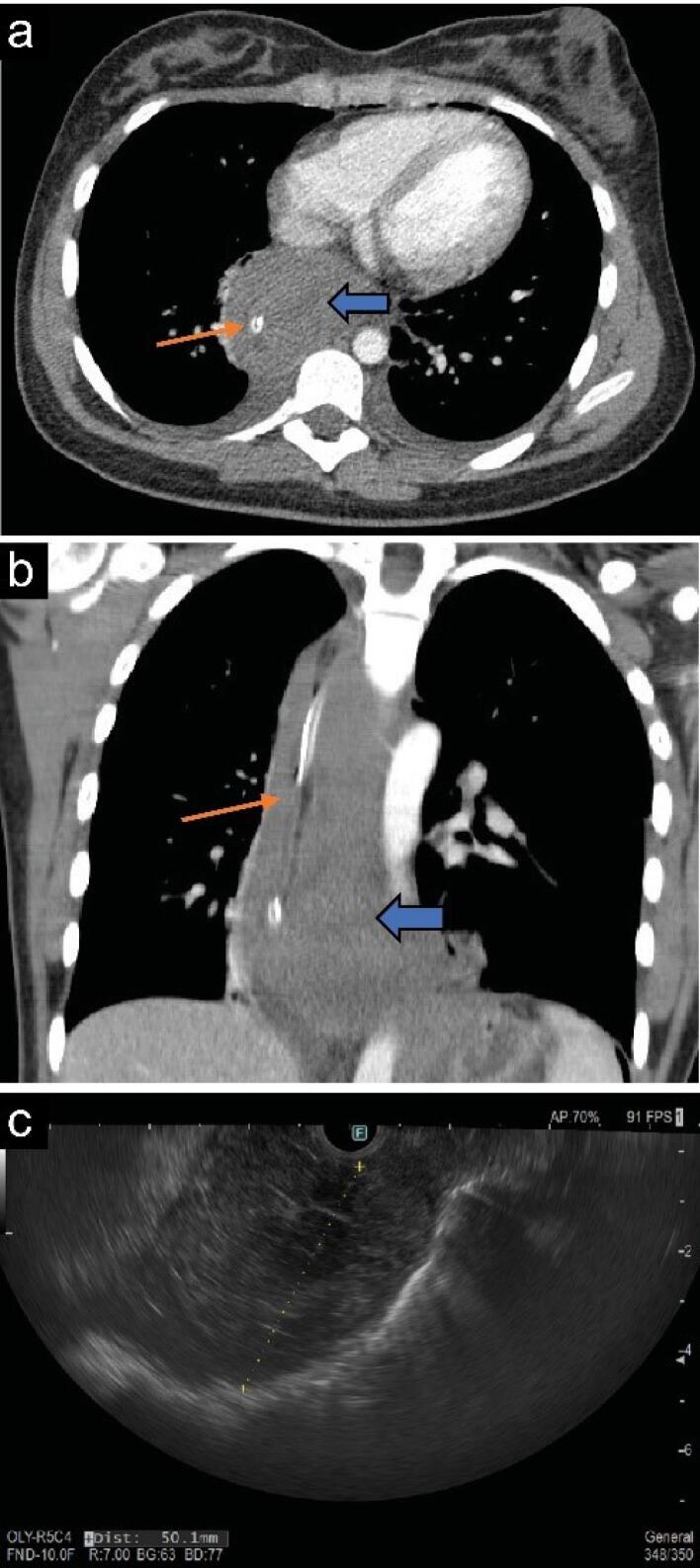
Axial (**a**) and (**b**) Coronal images in spiral chest CT scan with contrast show marked diffuse asymmetric circumferential wall thickening of the esophagus extending to the gastroesophageal junction (thick blue arrows in **a** and **b**). The nasogastric tube is visualized in the lumen of the esophagus (thin red arrows in **a** and **b**). (**c**) EUS showed a non-homogeneous mass in the esophageal wall originating from the muscularis propria, with no surrounding nodes.

**Figure 3:**
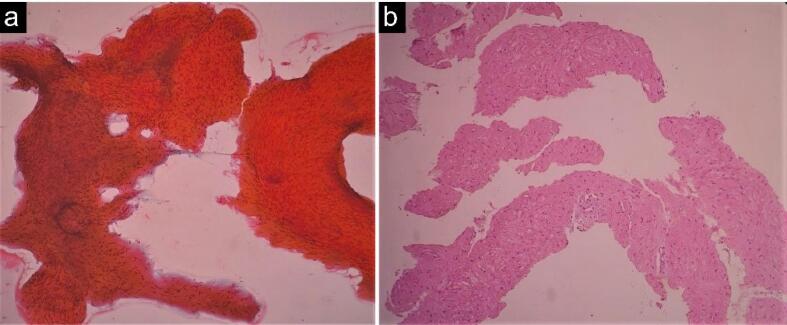
** (a) **Fine needle aspiration of the esophageal mass revealed a few clusters of benign-looking spindle cells (pap stain, × 100). **(b)** Needle biopsy from the esophageal mass shows a few cores of spindle smooth muscle cells with no significant atypia (Hematoxylin and eosin, × 100)

 A 16-year-old girl presented with progressive dysphagia and weight loss for the past 6 months. The general physical examination and laboratory findings were normal. Due to delayed passage of contrast and gastroesophageal junction (GEJ) narrowing on a time barium swallow, she was referred to our clinic for further evaluation regarding a possible motility disorder. On endoscopy, the mucosa of the esophagus appeared normal with spontaneous opening of the lower esophageal sphincter (LES). High-resolution water-perfused esophageal manometry detected the absence of peristalsis with panesophageal pressurization, but LES had repeatedly very high pressure with sudden drops (Figure 1). Chest computed tomography (CT) scan was requested, which revealed diffuse asymmetric circumferential wall thickening of the esophagus extending to the GEJ (Figure 2a, 2b). EUS showed a non-homogeneous mass in the esophageal wall originating from the muscularis propria, with no surrounding nodes. The lesion was extended from 30 to 27 cm from incisors in length and was 52 × 44.7 mm in cross section, at its maximum above the GEJ (Figure 2c). FNB under EUS-guidance was performed. Cytologic examination revealed a few clusters of spindle cells with uniform cells and no atypia. Histopathologic evaluation of the needle biopsy showed a few cores of spindle cells with abundant eosinophilic cytoplasm. There was no anaplasia or mitosis. Immunohistochemical study revealed immunoreactivity for smooth muscle actin, and no reactivity for C-kit, DOG-1, and S100. Proliferation index (ki-67) was low. The diagnosis of esophageal leiomyoma was confirmed (Figure 3).

 Surgical treatment was suggested for her, but she and her family refused.

## Discussion

 Esophageal leiomyoma is a very rare tumor that arises from mesenchymal tissue. It is the most common benign tumor of the esophagus.^4^

 More than 50% of the patients are asymptomatic and are diagnosed incidentally while others may present with dysphagia or epigastric pain.^2,4^ As these tumors grow away from the lumen, dysphagia might be a late symptom. The size of esophageal leiomyomas can vary, with reported sizes ranging from 1 to 29 cm.^4-6^ Large esophageal leiomyomas are uncommon and have been reported as follows: smaller than 5 cm in 49% of cases, 5 to 9 cm in 33.7%, 10 to 14 cm in 12.2%, 15 to 19 cm in 2.5%, and larger than 20 cm in 2.5% of reported cases.^4,6^ Leiomyomas larger than 10 cm are defined as giant leiomyomas.^4,5,7^ Dysphagia, retrosternal pain, and pyrosis are the most common symptoms.^4,7^ However, no relationship has been found between symptoms and the size or location of the tumor.^4,7^ Surgery is the cornerstone of treatment and typically involves extramucosal blunt enucleation. Esophagectomy may be necessary in the presence of giant leiomyomas.^1,8^ The most common indications of surgery include symptomatic patients, tumor larger than 3–5 cm, or rapidly increasing tumor size. It is recommended that asymptomatic patients with tumors smaller than 2 cm be followed periodically.^2,9^

 Our patient had a symptomatic giant leiomyoma, which is quite rare in adolescents. Our initial assumption was infiltrating malignant disease, which was ruled out by performing FNB twice and immunohistochemical staining of the obtained tissue.

 Esophageal leiomyoma should be considered even in adolescents with intramural lesions of the esophagus. Any decision should be based on tissue diagnosis, which is now possible with EUS-guided biopsies with minimal risk.

## Conclusion

 This case highlights the rare occurrence of large esophageal leiomyoma in an adolescent, presenting with progressive dysphagia and weight loss that initially mimicked achalasia. Despite its benign nature, the tumor’s size and symptomatic presentation underscore the importance of thorough evaluation and accurate tissue diagnosis. Endoscopic ultrasonography (EUS) combined with fine needle biopsy (FNB) proved essential in differentiating leiomyoma from malignant or other intramural esophageal lesions. Although surgical enucleation remains the standard treatment for symptomatic or large tumors, patient and family preferences must also be respected in clinical decision-making. Ultimately, this report emphasizes the need to consider esophageal leiomyoma in the differential diagnosis of intramural lesions, even in young patients, and highlights the role of minimally invasive diagnostic tools in guiding appropriate management.
